# Vitreous changes after intravitreal bevacizumab monotherapy for retinopathy of prematurity: a case series

**DOI:** 10.1186/s40942-018-0113-3

**Published:** 2018-03-19

**Authors:** Nasser Shoeibi, Seyedeh Maryam Hosseini, Touka Banaee, Mohammad-Reza Ansari-Astaneh, Majid Abrishami, Hamid Ahmadieh

**Affiliations:** 1 Eye Research Center, Mashhad University of Medical Sciences, Mashhad, Iran; 2 Retina Research Center, Khatam-Al-Anbia Eye Hospital, Mashhad University of Medical Sciences, Abutalib Junction, Kolahdouz Blvd, Mashhad, 9195961151 Iran; 3 Ophthalmic Research Center, Shahid Beheshti University of Medical Sciences, Tehran, Iran

**Keywords:** Anti-VEGF, Bevacizumab, Retinopathy of prematurity, Vitreous detachment, Vitreous condensation

## Abstract

**Purpose:**

Reporting a special clinical finding after intravitreal bevacizumab monotherapy for retinopathy of prematurity.

**Methods:**

In a retrospective case series, the clinical courses of five premature infants with similar vitreous changes after a single dose of intravitreal bevacizumab (IVB) injection without additional laser therapy were reported.

**Results:**

The mean post-conceptional age at IVB injection was 39.8 ± 2.2 (range 37–43) weeks. Localized vitreous syneresis and linear fibrotic vitreous condensation occurred 8.2 ± 2.3 weeks after IVB monotherapy in our patients (15.5% of injections). The mean last post injection visit was 61.6 ± 5.3 weeks (post-conceptional age). Further regression and complete retinal vascularization occurred in all patients.

**Conclusions:**

Thread-like vitreous condensation with localized vitreous liquefaction may be related to involutional ROP disease itself, combined to anti VEGF therapy and may be a predictor factor for further regression and retinal vascularization. The case series describes a successful response to anti-VEGF monotherapy with no further complications.

## Introduction

Retinopathy of prematurity (ROP) is a disorder of neonatal retinal vascularization. At present, ablation of peripheral avascular retina is the standard treatment method for type-I ROP [1]. There are several disadvantages to retinal laser photocoagulation including posterior synechiae, macular ectopia, and scarring of the peripheral retina and choroid, as well as the need for general anesthesia although some of these complications may be the natural course of the disease [2]. This is why a safe, effective and easy-to-apply treatment is still highly sought.

Elevated intraocular concentration of VEGF is consistently documented in ROP patients [3–5]. Thus, bevacizumab has been applied in ROP cases, with recent reports on both its efficacy and complications [3]. There is no consensus in the literature on the safety, efficacy, and appropriate indication of bevacizumab injection in patients with ROP. Although a retardation of normal vascularization is noted, IVB monotherapy has been considered by some to be a relatively safe and effective treatment modality for stage 3 ROP, including zone I cases [6]. Development of peripheral retinal vessels continues after administering intravitreal bevacizumab, whereas conventional laser therapy leads to permanent destruction of the peripheral retina.

In this paper, we reported the clinical courses, fundus photographs and specifications of nine eyes of five premature infants with a similar vitreous change (a linear fibrillar vitreous condensation resembling partial vitreous detachment) at the original fibrovascular ridge, developed after a single dose of intravitreal bevacizumab (IVB) for treating stage 3 with or without plus disease. To the best of our knowledge, this is the first report describing a special clinical finding after intravitreal bevacizumab monotherapy for retinopathy of prematurity.

## Methods

All of the patients who were referred to the ROP clinic (a tertiary referral specific clinic designed for ROP treatment and F/U in the east of our country), Khatam-Al-Anbia Ophthalmology Hospital, Mashhad, Iran, between May 2012 and June 2014 and received IVB for ROP were reviewed retrospectively and the patients with a medical record of linear fibrillar vitreous condensation, partial vitreous detachment at the site of original fibrovascular ridge or any other vitreous change after IVB injection were collected. Exclusion criteria were: patients with incomplete recordings or laser treatment after injection. All patients were examined by an experienced retina specialist. In each exam session, indirect ophthalmoscopy with scleral depression and fundus photography with RetCam III (Clarity Medical Systems Inc., Pleasanton, CA, USA) were performed. The patients with type 1 ROP in stage 3 were identified as eligible for IVB injection. In our referral center, IVB is the first line for ROP patients eligible for treatment and laser treatment is used for the cases not responsive to IVB or when recurrence occurs after one or two IVB injections. All of the reported cases in this series were treated with IVB monotherapy as the first line and no further treatment including laser was necessary. Following thorough and comprehensive explanations regarding the risks imposed by intravitreal injection as well as the off-label nature of the treatment, parents signed informed written consent forms, and the patients subsequently received a single dose of IVB (0.625 mg/0.025 ml, 1.75 mm from the limbus) in the operating room with topical anesthesia. They were then followed: the day after the operation for the first time and every 5–7 days thereafter. Partial vitreous detachment was confirmed by RetCam III and fundus examination. B-Scan ultrasonography (NIDEK Echoscan US-4000, Gamagori, Japan) was performed in one patient in order to document the presence of partial vitreous detachment. We did not evaluate vitreous changes in non-treated or laser-treated patients. We also din not compare these changes between treated and non-treated patients. This study was approved and qualified by ethics committee of Khatam-al-Anbia Eye Research Center and institutional review board of Mashhad University of Medical Sciences.

## Results

**Fig. 1:**
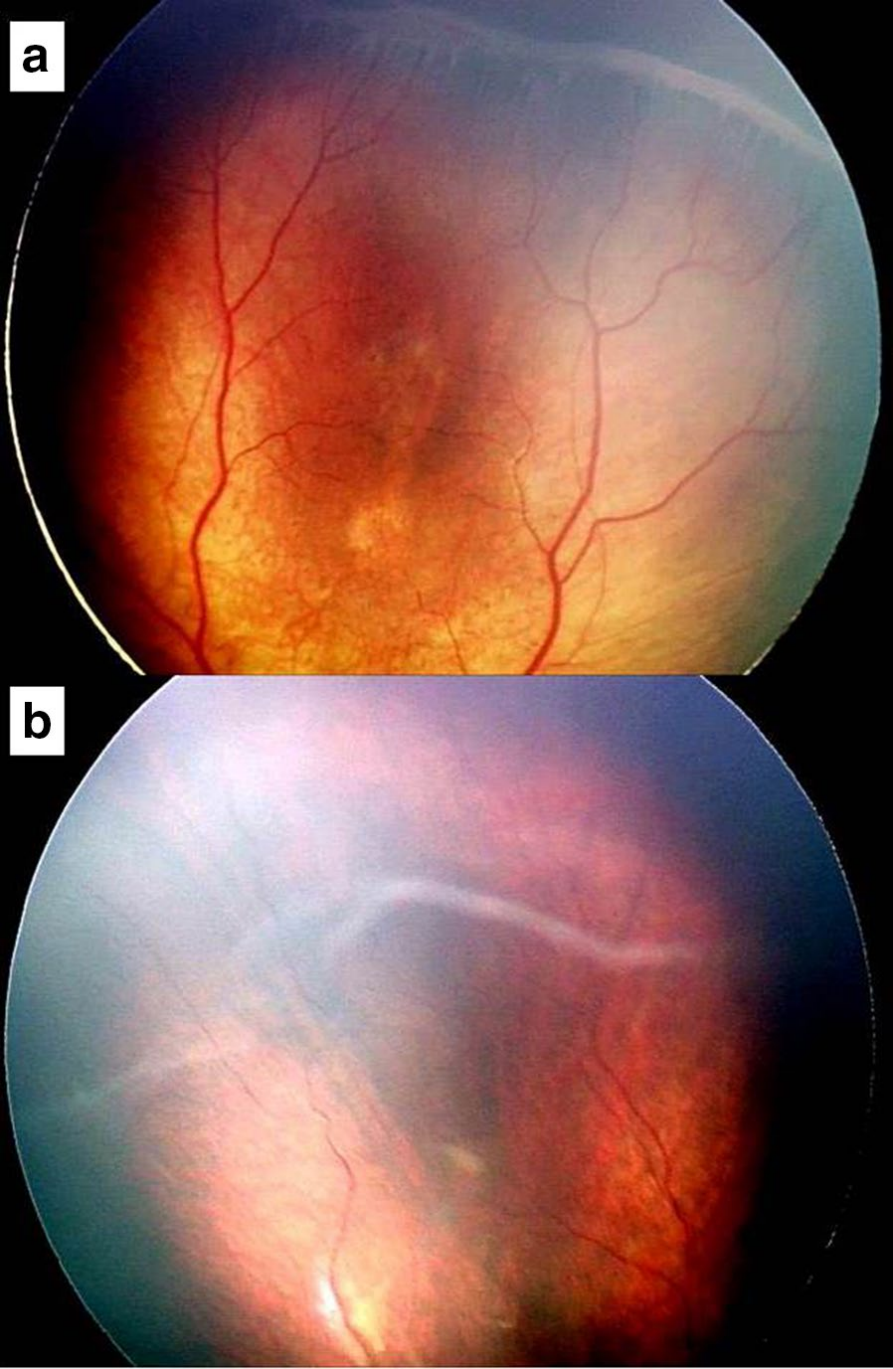
Fundus photography of case 1: Before treatment, fibrovascular ridge at temporal retina in posterior zone II (**a**). Seven months after the treatment, vitreous detachment and floating vitreous ridge with regressed retinal ridge and ingress of normal vasculature down to peripheral retina (**b**)

**Fig. 2:**
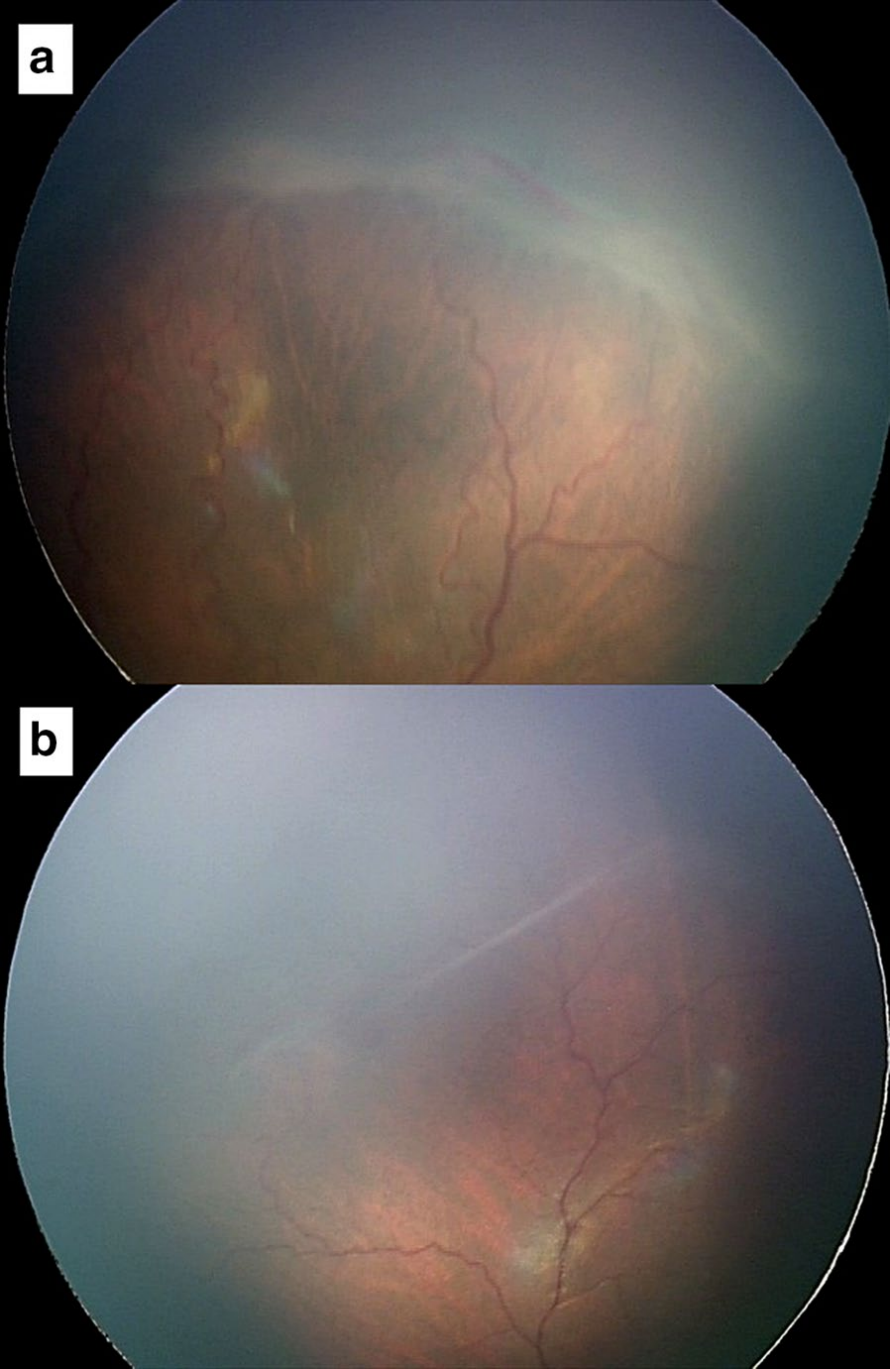
Fundus photography of case 5: Before treatment, stage 3 in zone II (**a**). At the last follow up, thread-like vitreous condensation with regressed retinal ridge and complete vascularization (**b**)

**Fig. 3:**
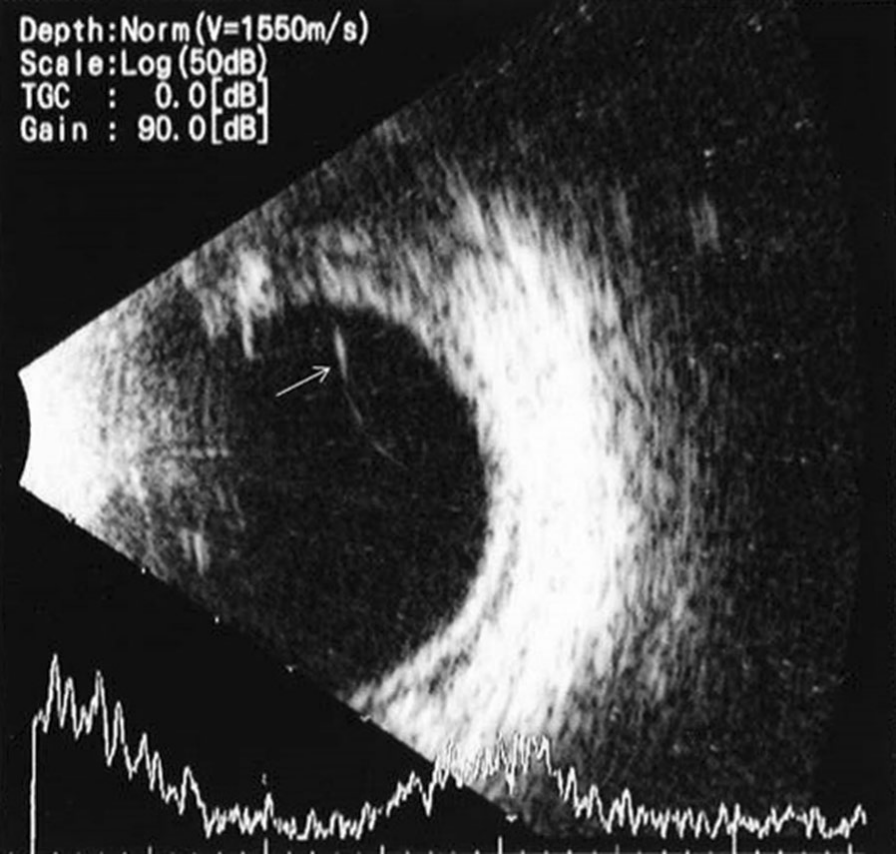
B-Scan ultrasonography of case 1. White arrow indicates the partial vitreous linear condensation

A total of 1124 patients (2248 eyes) with ROP referred to our clinic between May 2012 and June 2014 for further examination. Fifty-eight injections (2.58% of eyes) were submitted to intravitreal bevacizumab injection during this period. Nine eyes of five patients had the similar feature of vitreous changes after IVB injection (15.5% of injections). Clinical features and the patient characteristics are summarized in a Table 1. The mean postconceptional age at IVB injection was 39.8 ± 2.2 (range 37–43) weeks. Nine eyes developed similar vitreous changes including thread-like vitreous condensation with localized vitreous liquefaction or linear vitreous condensation. This process started after the regression of neovascularization at the site of temporal ridge. Presentation of the initial vitreous change was 8.2 ± 2.3 (range from 4 to 12 weeks) after IVB injection and the mean last post injection visit was 61.6 ± 5.3 weeks (post-conceptional age). Full peripheral retinal vascularization developed in all injected eyes. The original retinal ridge disappeared completely in all patients. Three weeks after IVB, small localized tractional retinal detachment (TRD) developed in one patient at the ridge site that resolved 4.5 weeks later. Seven weeks after IVB, a localized preretinal hemorrhage was observed at the posterior border of the temporal ridge which resolved in 2 weeks in another patient. The vitreous changes were bilateral in all five patients except case No 4. No further complication was observed until the last followed up. Complete retinal vascularization and complete regression of the ridge occurred in all patients. Fundus photograph of the patient No. 1 and 5 and B scan ultrasonographic picture of the patient No. 1 are presented in Figs. 1, 2 and 3, respectively.

**Table 1:** Clinical characteristics of the studied patient

Case no.	1		2		3		4		5		Mean ± SD
	OD	OS	OD^1^	OS	OD	OS	OD	OS	OD^2^	OS	
Postconceptional age at birth (weeks)	24	24	31	31	31	31	32	32	29	29	29.4 ± 3.2
Birth weight (grams)	1040	1040	1480	1480	1300	1300	1700	1700	1100	1100	1324.0 ± 272.5
Zone of ROP	2	2	1	1	2	2	2	2	2	2	–
Stage of ROP	3	3	3	3	3	3	3	3	3	3	–
Plus disease	No	Yes	Yes	Yes	No	Yes	Yes	No	Yes	Yes	
Vitreous changes	Yes	Yes	Yes	Yes	Yes	Yes	No	Yes	Yes	Yes	–
Post-conceptional age at IVB injection (weeks)	37	37	40	40	40	40	38	39	43	43	39.8 ± 2.2
Time of starting regression after IVB injection (weeks)	4	4	2	2	4	4	2	2	3	3	3.0 ± 1.1
Presentation of the initial	6	8	10.5	7.5	9	12	–	8	9	4	8.2 ± 2.3
Vitreous changes (weeks After injection) last follow up (weeks)	69	69	65	65	59	59	59	59	56	56	61.6 ± 5.3

^1^Three weπeks after IVB, localized TRD developed at ridge site that resolved 4.5 weeks afterward

^2^Seven weeks after IVB, a localized preretinal hemorrhage was observed at the posterior border of the temporal ridge which resolved in 2 weeks

## Discussion

This study presents the similar vitreous change at the temporal retinal ridge of ROP following IVB injection in nine eyes of five patients. All eyes in our study had extensive liquefaction with linear vitreous condensation overlying the ridge; changes such as these are quite rare in eyes that are not treated with IVB. Our described vitreous changes are similar to those demonstrated by Foos that happen in the chronic phase of stage 3 in the vitreous overlying the ridge [7]. Foos described some changes in the vitreous overlying the ridge including degradation, condensation, and liquefaction of the vitreous collagen. This can result in the vitreous overlying the ridge becoming opalescent with linear condensation. They could not explain the importance of these changes in regressing or progressing the ROP. We suppose that a similar process can be induced by IVB injection in some ROP patients.

The vitreoretinal interface (VRI) is a complex composite structure connecting the vitreous cortex to the inner retina. As the vitreous ages, there is gel liquefaction and the development of fluid-filled pockets, typically beginning in the central vitreous cavity. This leads to posterior vitreous detachment with time [8]. Anti-VEGF agents have some effects on vitreous and vitreoretinal interface properties. Intravitreal anti-VEGF treatment causes increased fibrosis by increasing the level of connective tissue growth factor (CTGF) [9]. The shift in the balance between levels of CTGF and VEGF in the eye is associated with this angiofibrotic switch. CTGF activates TGF-β signals by direct binding in the extracellular space and TGF-β stimulates the differentiation of fibroblasts (or protomyofibroblasts) into myofibroblasts that are characterized by expression of α-smooth muscle actin. Perhaps, this mechanism is involved in the complex process of TGF-β-mediated matrix contraction [10]. Acute deterioration of tractional retinal detachment (TRD) following intravitreal injection of bevacizumab has been reported in some patients [11]. Honda et al. also reported progression of TRD after IVB injection in one ROP infant [12]. This may explain our described fibrotic linear changes in vitreous of IVB- treated patients. On the other hand, it has been reported that about 5% of adult patients may develop PVD within 1 month after a single IVB injection [13]. Detachment usually occurs after multiple injections, beginning at the posterior pole. In our series, a linear vitreous fibrotic condensation resembling an incomplete and localized vitreous syneresis or partial vitreous detachment occurred in nine eyes. This process started after the regression of neovascularization at the site of temporal ridge.

Rouvas et al. reported spontaneous vitreous detachment and resolution of vitreomacular traction following ranimizumab injection [14]. The authors suggested that the pathophysiological mechanism is based on a dual combined effect: a mechanical effect, whereby an intravitreal injection causes vitreous liquefaction, and an increase of vitreous volume, and a functional effect arising from the anti-VEGF-induced retinal thickness reduction. In our patients, the same mechanism of volume effect and a functional effect arising from the anti-VEGF-induced shrinkage of regressed vessels and ROP ridge can be postulated.

Hikichi et al. demonstrated that eyes with cicatricial ROP that had undergone cryopexy and/or photocoagulation had vitreous changes such as marked vitreous liquefaction, vitreous membranes traversing the liquefied vitreous cavity, and the low incidence of PVD despite advanced liquefaction [15]. The clinical changes described in our patients are similar to these findings in the vitreous liquefaction and fibrous proliferation in vitreous cavity.

We did not observe any important complication in our series. In our series, a temporary small localized TRD occurred in one eye 3 weeks after the injection, which resolved in 1.5 weeks. In another eye, temporary preretinal hemorrhage was seen, indicating the probable presence of focal tractions in the process of separation of the vitreous from retina. Lee et al. reported a series of three patients with the delayed onset of atypical fibrous traction membrane arising along the major vascular arcades, with a latency period of 2.5–4 months after IVB injection [16]. The traction finally progressed to tractional retinal detachment (TRD) in three out of the five eyes. In Lee’s series, two patients had a history of concurrent laser therapy. The third patient received two IVB injections. In our series, no case received laser treatment, and IVB injection was not repeated. This may be the reason of low complications in our series.

The mean post-conceptional age for intravitreal bevacizumab injection (IVB injection) described in our series was later compared to data found in the literature which is around 37 weeks post conceptional age [17]. Fortes et al. concluded that inborn patients were treated for retinopathy of prematurity during the week 37 of post-conceptional age while transferred patients were treated, usually, after week 39 [18]. In our series, 6 eyes (out of 9) were treated in due date (PMA 40 weeks) or later. Our center is a referral in the east of our country and this may explain the later post-conceptional age of the treatment in our series. On the other hand, this may also affect the vitreous condensation found in this case series.

To the best of our knowledge, this is the first report of vitreous changes following IVB injection in ROP patients. Close follow-up is strongly recommended after this intervention for stage 3+ ROP, even if the disease seems to be regressing. According to our described clinical features, it is of paramount significance to follow up patients for possible development of retinal detachment or breaks after IVB for ROP. On the other hand, once vitreous changes and full retinal vascularization occur after IVB injection, they may potentially be regarded as a protective factor against the late complications of ROP.

We also observed this clinical finding in a very small population of non-treated ROP patients. We did not evaluate or compare the clinical features or incidence of these changes in non-treated or laser-treated patients, but according to our experience, vitreous changes were most marked in the IVB treated patients, so we concluded that this clinical finding most likely results from the disease itself and the treatment. We also did not explain the influence of anatomical variations such as the globe size or other confounding factors such as age, sex, etc. in the rate of vitreous changes after IVB injection. Further investigations with a longer follow-up and a larger sample size seem warranted in this field. Prognosis of the above-mentioned vitreous changes after IVB injection needs further investigation. As all of our patients had regressed ROP with complete retinal vascularization, this finding could be considered a good prognostic factor for ROP regression.

## Conclusions

The vitreous findings described (thread-like vitreous condensation with localized vitreous liquefaction or linear vitreous condensation) may be related to involutional ROP disease itself, combined with anti-VEGF therapy and possibly influenced the treatment near due date and may be a predictor factor for further regression and retinal vascularization. The case series describes a successful response to anti-VEGF monotherapy with no further complications such as disease reactivation or progression to retinal detachment.
